# Misdiagnosis analysis of 2291 cases of haematolymphoid neoplasms

**DOI:** 10.3389/fonc.2023.1128636

**Published:** 2023-04-26

**Authors:** Jing Deng, Xiaona Zuo, Liuyi Yang, Zifen Gao, Chunju Zhou, Ligai Guo

**Affiliations:** ^1^ Department of Pathology, Beijing GoBroad Boren Hospital, Beijing, China; ^2^ Department of Pathology, Peking University Third Hospital, Beijing, China; ^3^ Department of Pathology, Beijing Children's Hospital, Capital Medical University, Beijing, China

**Keywords:** Haematolymphoid, lymphoma, pathology, misdiagnosis, diagnostic pitfalls, expert review, accurate diagnosis

## Abstract

**Objective:**

To retrospectively analyze the reasons for misdiagnosis of haematolymphoid neoplasms and provide experience for improving the diagnostic level in China.

**Methods:**

A retrospective analysis was performed on 2291 cases of haematolymphoid diseases evaluated by the Department of Pathology of our hospital from 1 July 2019 to 30 June 2021. All 2291 cases were reviewed by two hematopathologist experts and classified according to the 2017 revised WHO classification criteria, supplemented immunohistochemistry (IHC), molecular biology and genetic information as needed. The diagnostic discordance between primary and expert review was evaluated. The possible causes of the diagnostic discrepancies were analyzed for each step involved in the procedure of diagnosis.

**Results:**

In total, 912 cases did not conform to the expert diagnoses among all the 2291 cases, with a total misdiagnosis rate of 39.8%. Among them, misdiagnosis between benign and malignant lesions accounted for 24.3% (222/912), misdiagnosis between haematolymphoid neoplasms and non-haematolymphoid neoplasms accounted for 3.3% (30/912), misdiagnosis among lineages accounted for 9.3% (85/912), misclassification in lymphoma subtypes accounted for 60.8% (554/912), and other misdiagnoses among benign lesions accounted for 2.3% (21/912) of cases, among which misclassification of lymphoma subtypes was the most common.

**Conclusion:**

The accurate diagnosis of haematolymphoid neoplasms is challenging, involving various types of misdiagnosis and complicated causes, however, it is important for precise treatment. Through this analysis, we aimed to highlight the importance of accurate diagnosis, avoid diagnostic pitfalls and to improve the diagnostic level in our country.

## 1 Introduction

The pathological diagnosis of haematolymphoid neoplasms has always been challenging in clinical pathology. In addition, clinical treatment regimens are different for distinct subtypes, leading to a significantly different prognosis (1). To achieve standardized, precise and individualized therapeutic approaches, it is critical to make a precise pathological diagnosis. The overall misdiagnosis rate of haematolymphoid neoplasms is relatively higher (2–6), and the documented rates are as high as 27.3% (3) after the adoption of the World Health Organization (WHO) Classification in the year 2001. Moreover, the expert review was emphasized in the literatures. Recently, a large-sample study in France was carried out with a misdiagnosis rate of up to 19.7% (6). However, there is a lack of related studies in China. Hence, a retrospective analysis of the misdiagnosis of haematolymphoid neoplasms in China is of great significance.

As a specialized hospital for refractory/recurrent haematolymphoid tumors, Beijing GoBroad Boren Hospital established its Department of Pathology on 1 July 2019. By 30 June 2021, the Department of Pathology received consultations of 2291 cases related to haematolymphoid diseases. According to the 2017 revised WHO Classification of Tumors of Haematopoietic and Lymphoid Tissues, we investigated all procedures, including acquisition of clinical information, tissue sampling, fixation, hematoxylin-eosin (HE) and immunohistochemical staining (IHC), interpretation of HE and IHC results, supplemental data such as flow cytometry (FCM), molecular biology and cytogenetics, and pathologists’ comprehension of the diagnostic criteria, and analyzed possible causes of diagnostic discrepancies to provide references for the diagnosis of haematolymphoid neoplasms and improve the accuracy of pathological diagnosis.

## 2 Materials and methods

### 2.1 General data

A total of 2291 consult cases related to haematolymphoid diseases in the Department of Pathology of our hospital from 1 July 2019 to 30 June 2021 were analyzed, whose samples were reviewed by outside pathologists first from nearly 600 hospitals across the country. There were 1365 males and 926 females aged from 11 days to 91 years. All samples were primarily diagnosed by outside pathologists first, then reviewed by two hematopathologist experts, and finally diagnozed by experts according to the 2017 revised WHO classification criteria.

### 2.2 Detection method

The received materials contained original slides (HE and IHC) and/or unstained slides or formalin-fixed paraffin-embedded block(s) as well as the clinical information (including tissue site, gender, age, symptoms, and imaging material). The following IHC staining was performed using an automatic IHC staining machine (Leica BOND-MAX). Primary antibodies were purchased from Beijing Zhongshan Golden Bridge Biotechnology Co., Ltd., and Gene Tech (Shanghai) Company Limited. EBV-encoded small RNAs (EBER) were detected by *in situ* hybridization (ISH), and the probes and corresponding kits were purchased from Leica Biosystems Nussloch GmbH. All operations were carried out according to the manufacturer’s instructions. The gene rearrangement of MYC, BCL2, BCL6, and CCND1 was detected using fluorescence *in situ* hybridization (FISH). The probe and kit were purchased from Guangzhou LBP Medicine Science & Technology Co., Ltd. The immunoglobulin (IG) and T-cell receptor (TCR) rearrangement were detected by polymerase chain reaction (PCR) based on the BIOMED-2 standardized clonality analysis system.

## 3 Results

**Figure 1:**
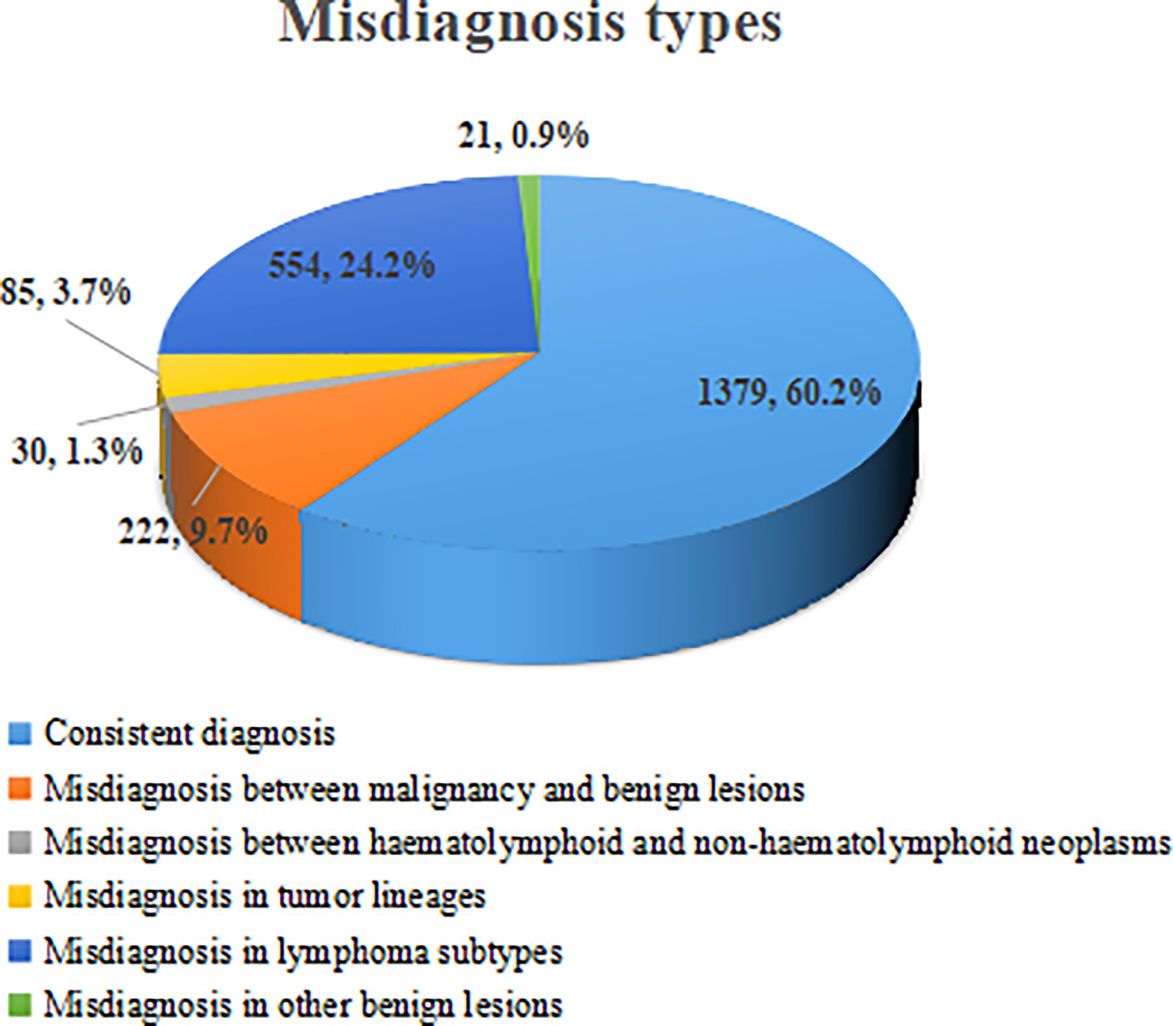
Misdiagnosis analysis of 2291 cases.

Among 2291 cases, 912 were misdiagnosed, and the overall misdiagnosis rate was 39.8%. Among these cases, the misdiagnosis between malignancy and benign lesions accounted for 9.7% (222/2291), the misdiagnosis between haematolymphoid neoplasms and non-haematolymphoid neoplasms accounted for 1.3% (30/2291), the misdiagnosis between tumor lineages accounted for 3.7% (85/2291), the misdiagnosis between lymphoma subtypes accounted for 24.2% (554/2291), and the misdiagnosis of other benign lesions accounted for 0.9% (21/2291). Misdiagnosis of lymphoma subtypes was the most frequent (**Figure 1**).

Among 2291 cases, there were 1134 cases of non-Hodgkin B-cell neoplasms (B-NHL), including diffuse large B-cell lymphoma (DLBCL), Burkitt lymphoma (BL), high-grade B-cell lymphoma (HGBL), follicular lymphoma (FL), small B-cell lymphoma/chronic lymphocytic leukemia (SLL/CLL), marginal zone B-cell lymphoma (MZL), lymphoplasmacytic lymphoma (LPL), mantle cell lymphoma (MCL), plasmablastic lymphoma (PBL), B-lymphoblastic lymphoma/leukemia (B-LBL/ALL), and plasmacytomas (PCN). Of all the B-NHL, there were 418 cases of DLBCL, accounting for the highest proportion (18.3%, 418/2291), including diffuse large B-cell lymphoma-not otherwise specified (DLBCL-NOS), primary mediastinal large B-cell lymphoma (PMBL), EBV-positive DLBCL and ALK-positive large B-cell lymphoma (LBCL). Among the 418 cases of DLBCL, 254 cases (60.8%, 254/418) had a concordance diagnosis; 24 cases had a misdiagnosis between DLBCL and low-grade B-cell lymphoma (total misdiagnosis rate: 1.1%, 24/2291), and 22 cases had a misdiagnosis between DLBCL and grade 3A FL (1.0%, 22/2291). Among 128 cases of BL, the diagnostic concordance rate was 79.7% (102/128). Among 88 cases of HGBL, the diagnostic concordance rate was only 15.9% (14/88). There were 29 cases of HGBL who were mis-diagnosed as BL, including 4 cases without FISH tests, 12 cases with strong positive BCL2, 13 cases with no MYC rearrangement. Among the 6 cases of HGBLs with MYC and BCL2 and/or BCL6 rearrangements (double-hit HGBLs), 5 cases were undiagnosed for lack of FISH tests, and 1 case was diagnosed as DLBCL with both MYC and BCL6 rearrangements. Among 90 cases of high-grade FL (grade 3A and 3B), the diagnostic concordance rate was 32.2% (29/90), 19 cases were primarily diagnosed as DLBCL, 8 cases were primarily diagnosed as low-grade FL (grade 1-2) and 20 cases had misdiagnoses between high-grade and low-grade FL (grade 1-2) among the 2291 cases (0.9%, 20/2291). Among 249 cases of small B-cell lymphoma (excluding MCL), 141 cases (56.6%) had consistent diagnoses. Among 24 cases of MCL, 19 cases (79.2%) had consistent diagnoses. Among 70 cases of B-LBL/ALL, 9 cases were misdiagnosed as mature B-NHL (12.9%, 9/70), and misdiagnosis between B-LBL/ALL and mature B-NHL occurred in 14 cases of all the 2291 cases (0.6%, 14/2291).

There were 424 cases of T/NK-cell tumors, including T-lymphoblastic lymphoma/leukemia (T-LBL/ALL) (98 cases), anaplastic large cell lymphoma (ALCL) (98 cases), NK/T-cell lymphoma (45 cases), EBV-positive T/NK-cell proliferative disease (31 cases), angioimmunoblastic T-cell lymphoma (AITL) (52 cases), peripheral T-cell lymphoma-not otherwise specified (PTCL-NOS) and other mature T-cell non-Hodgkin lymphoma (T-NHL) (99 cases), with diagnostic coincidence rates of 84.7%, 67.4%, 62.2%, 58.1%, 55.8%, and 38.4%, respectively. Excluding T-LBL/ALL, mature T/NK-cell neoplasms had a relatively lower concordance (180/326, 55.2%).

There were 195 cases of classic Hodgkin lymphoma (CHL), including 61 misdiagnosed cases (31.3%). Among 61 misdiagnosed cases, 14 cases were misdiagnosed as benign hyperplasia, 3 cases were misdiagnosed as non-haematolymphoid neoplasms, 13 cases were misdiagnosed as T-cell lymphomas, 2 cases were misdiagnosed as histiocytic neoplasms, 1 case was misdiagnosed as myeloid neoplasms, 10 cases were misdiagnosed as DLBCL (including 2 cases of EBV-positive DLBCL, 2 cases of T-lymphocyte/histiocyte-rich large B-cell lymphoma), 7 cases were misdiagnosed as B-cell lymphoma-unclassified with features between diffuse large B-cell lymphoma and classic Hodgkin lymphoma (DLBC-U, grey zone lymphoma), 3 cases were misdiagnosed as nodular lymphoma primary Hodgkin’s lymphoma (NLPHL), and 8 cases had no definite results in the initial diagnosis. The differential diagnosis between CHL and T-NHL sometimes can be difficult, and PCR-TCR was performed in 4 cases who were primarily diagnosed as AITL.

Mixed phenotype acute leukemia (MPAL) and blast plasmacytoid dendritic cell neoplasm (BPDCN) were rare subtypes of myeloid and histiocytic/dendritic neoplasms, unsurprisingly, with the highest misdiagnosis rates (9/10, 90%; 7/8, 87.5%; respectively). For MPAL, 4 cases were misdiagnosed as T-LBL/ALL, 1 case was misdiagnosed as PTCL, 2 cases were misdiagnosed as B-LBL/ALL, and 2 cases were misdiagnosed as myeloid sarcoma. The diagnosis of MPAL in extramedullary tissue is more challenging, and adequate immunostains and accurate interpretation are necessary. Meanwhile, FCM is of great significance. Two cases were finally diagnosed referring to the subsequent FCM results of bone marrow.

There were 48 cases of non-haematolymphoid neoplasms, of which 13 were misdiagnosed as haematolymphoid neoplasms, with a diagnosis discordance rate of 27.1%. Misdiagnosis between haematolymphoid and non-haematolymphoid neoplasms occurred in 30 cases of all the 2291 cases, and the most commonly involved non-haematolymphoid neoplasms were poorly differentiated carcinomas, sarcoma, neuroendocrine tumors and thymoma.

There were 399 cases of benign hyperplasia (including Castleman disease), including 135 misdiagnosed cases, with a diagnosis discordance rate of 33.8%. Among 135 misdiagnosed cases, 118 cases were misdiagnosed malignant tumor. Notably, 222 out of 2291 cases in this study were misdiagnosed between benignancy and malignancy (that is, submitted diagnosis benign→expert diagnosis malignant, and vice versa) (accounting for 9.7% of the total cases and 24.2% of the total misdiagnosed cases).

Notably, among 554 cases of subtype misdiagnosis, 206 cases (nearly 40%) were only diagnosed as B-NHL or T-NHL at the initial diagnosis, with no clear subtypes. Most of these cases were from hospitals in less developed areas or with lower level, the IHC antibodis were limited and detection means such as FISH, FCM and molecular studies were not available in those laboratories. What’s more, beyond the technology, pathologists’ knowledge and practical experience can also make a big difference in the diagnosis.

The distribution of disease types and the analysis of misdiagnosis types are shown in **Tables 1**, **2**.

**Table 1:** Comparison of initial and expert diagnoses in 2291 cases.

Type	Expert diagnoses (cases)	Diagnostic concordance	
		(cases)	(%)
B-cell Neoplasms	1347	791	58.7
DLBCL	418	254	60.8
HGBL	88	14	15.9
BL	128	102	79.7
Small ;B-cell lymphoma (including grade 1-2 FL, SLL/CLL, MALToma,MZL and LPL)	249	141	56.6%
MCL	24	19	79.2%
Grade 3 FL	90	29	32.2%
PBL	4	1	25.0%
PCM	32	25	78.1%
B-LBL/ALL	70	50	71.4%
Invasive mature B-cell lymphoma (no clear subtype)	31	19	61.3%
CHL	195	134	68.7%
NLPHL	4	1	25.0%
DLBC-U (grey zone lymphoma)	14	2	14.3%
T/NK-cell Neoplasms	424	263	62.0%
PTCL, NOS	52	22	42.3%
AITL	52	29	55.8%
ALCL	98	66	67.4%
Other mature T-cell lymphomas	47	16	34.0%
Chronic NK/T-cell proliferative disease	1	1	100.0%
NK/T-cell lymphoma	45	28	62.2%
EBV-positive lymphoproliferative disease	31	18	58.1%
T-ALL/LBL	98	83	84.7%
Myeloid and Histiocytic/Dendritic Neoplasms	64	21	32.8%
Myeloid neoplasms	38	14	36.8%
MPAL	10	1	10.0%
BPDCN	8	1	12.5%
Histiocytic/dendritic cell neoplasms	8	5	62.5%
GVHD	4	1	25.0%
HLH	5	4	80.0%
Benign Disease	399	264	66.2%
Castleman disease	52	31	59.6%
Benign hyperplasia (lymphoid tissue)	347	233	67.2%
Non-haematolymphoid Neoplasms	48	35	72.9%
Total	2291	1379	60.2%

DLBCL, diffuse large B-cell lymphoma; HGBL, high-grade B-cell lymphoma; BL, Burkitt lymphoma; FL, follicular lymphoma; SLL/CLL, small B-cell lymphoma/chronic lymphocytic leukemia; MALToma, extranodal marginal zone lymphoma of mucosa-associated lymphoid tissue; MZL, marginal zone B-cell lymphoma; LPL, lymphoplasmacytic lymphoma; MCL, mantle cell lymphoma; PBL, plasmablastic lymphoma; PCN, plasmacytoma; B-LBL/ALL, B-lymphoblastic lymphoma/leukemia; CHL, classic Hodgkin lymphoma; NLPHL, nodular lymphocyte predominant Hodgkin lymphoma; PTCL, peripheral T-cell lymphoma; AITL, angioimmunoblastic T-cell lymphoma; ALCL, anaplastic large cell lymphoma; T-LBL/ALL, T lymphoblastic lymphoma/leukemia; MPAL, mixed phenotype acute leukemia; BPDCN, blast plasmacytoid dendritic cell neoplasm; GVHD, graft-versus-host disease; HLH, hemophagocytic lymphohistiocytosis; DLBC-U, (grey zone lymphoma) unclassified B-cell lymphoma with features that are intermediate between diffuse large B-cell lymphoma and classic Hodgkin lymphoma.

**Table 2:** Analysis of the misdiagnosis classifications.

Misdiagnosis types	Cases	Proportion of all misdiagnosed cases (n=912)	Proportion of all cases (n=2291)
1. Misdiagnosis between benignancy and malignancy	222	24.3%	9.7%
Benign lesion misdiagnosed as malignancy	119	13.1%	5.2%
Malignancy misdiagnosed as a benign lesion	103	11.3%	4.5%
2. Misdiagnosis between haematolymphoid and non-haematolymphoid neoplasms	30	3.3%	1.3%
Haematolymphoid neoplasm misdiagnosed as non-haematolymphoid neoplasm	19	2.1%	0.8%
Non-haematolymphoid neoplasm misdiagnosed as haematolymphoid neoplasm	11	1.2%	0.5%
3. Misdiagnosis between tumors from different lineage	85	9.3%	3.7%
Misdiagnosis between B-NHL and T/NK-cell neoplasms	17	1.9%	0.7%
Misdiagnosis between lymphoid and myeloid neoplasms	16	1.8%	0.7%
Misdiagnosis between lymphoid/myeloid neoplasms and MPAL	12	1.3%	0.5%
Misdiagnosis between T-NHL and CHL	25	2.7%	1.1%
Misdiagnosis between T-NHL and NLPHL	1	0.1%	0.04%
Misdiagnosis between T-NHL and NK-cell neoplasms	3	0.3%	0.1%
Misdiagnosis between lymphoid/myeloid neoplasms and BPDCN	6	0.7%	0.3%
Misdiagnosis between lymphoid neoplasms and histiocytic neoplasms	5	0.6%	0.2%
4. Misdiagnosis between subtypes	554	60.8%	24.2%
Misdiagnosis between DLBCL and small B-cell lymphoma (excluding MCL)	24	2.6%	1.1%
Misdiagnosis between DLBCL and FL-3A	22	2.4%	1.0%
Misdiagnosis between DLBCL and FL-3B	2	0.2%	0.1%
Misdiagnosis between other large B-cell lymphoma subtypes	5	0.6%	0.2%
Misdiagnosis between FL (grade 1-2) and FL (grade 3)	20	2.2%	0.9%
Misdiagnosis between FL-3A and FL-3B	5	0.6%	0.2%
Misdiagnosis between BL and other mature B-NHL	52	5.7%	2.3%
Misdiagnosis between HGBL and other mature B-NHL	77	8.4%	3.4%
Misdiagnosis between MCL and other mature B-NHL	10	1.1%	0.4%
Misdiagnosis between other small B-cell lymphomas (excluding MCL)	4	0.4%	0.2%
Misdiagnosis between mature B-NHL and B-LBL/ALL	14	1.5%	0.6%
Misdiagnosis between PCN and B-NHL	6	0.7%	0.3%
Misdiagnosis between CHL and B-NHL	17	1.9%	0.7%
Misdiagnosis between NLPHL and B-NHL	3	0.3%	0.1%
Misdiagnosis between CHL and NLPHL	3	0.3%	0.1%
Misdiagnosis between grey zone lymphoma and CHL/B-NHL	23	2.5%	1.0%
Misdiagnosis between NK/T-cell lymphoma and T-NHL	11	1.2%	0.5%
Misdiagnosis between AITL and other mature T-NHL	23	2.5%	1.0%
Misdiagnosis between ALCL and other mature T-NHL	10	1.1%	0.4%
Misdiagnosis between other mature T-cell tumor subtypes	12	1.3%	0.5%
Misdiagnosis between mature T-NHL and T-LBL/ALL	5	0.6%	0.2%
Clear subtype determined in the review but not in the initial diagnosis	206	22.6%	9.0%
5. Others	21	2.3%	0.9%
Misdiagnosis between Castleman disease and other benign diseases	17	1.9%	0.7%
Misdiagnosis between GVHD and chronic inflammation	3	0.3%	0.1%
Undiagnosed HLH	1	0.1%	0.04%

## 4 Discussion

The overall misdiagnosis rate was 39.8% in our study, indicating that the diagnostic efficacy of haematolymphoid neoplasms in China still lags behind compared to other studies (2–6). This study analyzed the reasons for misdiagnosis from the following aspects.

### 4.1 Tissue sampling and processing

High-quality tissue specimens and HE-stained sections are essential for pathological diagnosis, especially for haematolymphoid neoplasms. Because of the unique organizational structure of lymphoid tissue, the quality requirements for its slices are relatively higher than that for other types of samples.

#### 4.1.1 Tissue sampling

To ensure diagnostic accuracy, an excisional biopsy in lymph nodes is preferred (7). For ultrasound-guided core-needle biopsy, it is recommended to obtain at least 3 representative tissues with a core needle no smaller than 18 gauge (G) to ensure enough specimens for subsequent procedures, including HE, IHC, FCM and molecular testing (8).

#### 4.1.2 Tissue fixation

**Figure 2:**
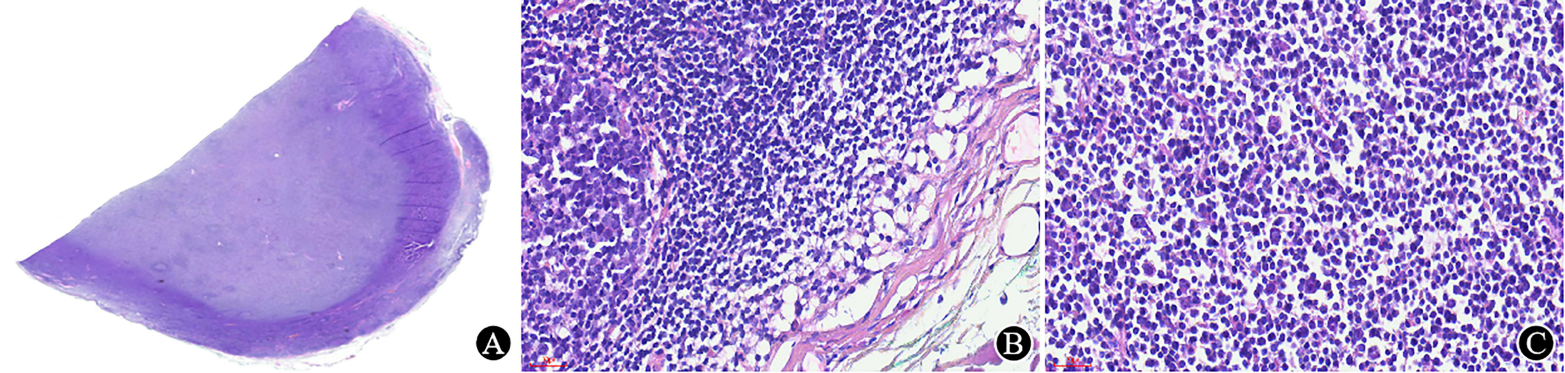
Poorly fixed lymph node. **(A)** Under low magnification, the lymph nodes is well stained in the periphery and lightly stained in the center (×5.5); **(B)** The structure near the capsule is well preserved, and the cell morphology is good (×400); **(C)** The tissue is dissociated, and the cells are shrunken in the area away from the capsule (×400).

The tissue should be fixed in 10% neutral formalin solution timely (within 30 minutes). Notably, lymph nodes should be dissected before fixation because the fibrous capsule of the lymph node can resist the penetration of the fixative, resulting in poor fixation. Furthermore, tissue that is poorly fixed can interfere with morphological observation (**Figure 2**), IHC staining results, and even molecular tests.

#### 4.1.3 Tissue sectioning

**Figure 3:**
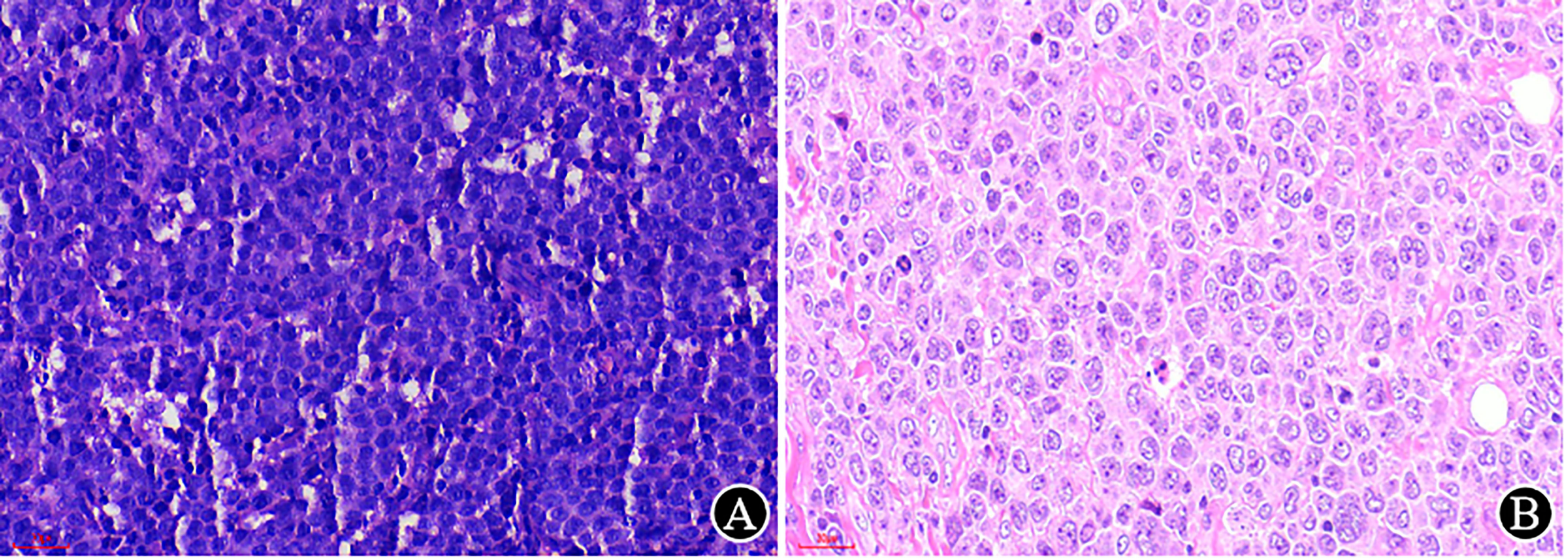
The impact of the thickness of the HE section on morphological observation (×400). **(A)** The previously prepared slice (at another hospital) is thick, with a medium cell volume and unclear structure; **(B)** The reprepared slice (at our hospital) is thin, with a clear cellular detail and larger volume.

High-quality HE sections are the basis of pathological diagnosis. The quality of HE sections depends on each step of tissue processing, and a thickness of 2-4 μm is preferred (7). In our laboratory, a 3 μm slice is required for both HE and IHC, which can clearly display the tissue structure and cellular detail (**Figure 3**).

### 4.2 Immunohistochemical staining

Immunohistochemistry plays an important role in the accurate diagnosis and definition of disease subtypes in hematopathology. Of all the 912 misdiagnosed cases in this study, IHC is always rechecked or performed significant additional IHC staining. The related errors in this procedure have been analyzed in the literature (9). In our study, the IHC-related errors were discussed from the following two aspects:

#### 4.2.1 Insufficient application of antibodies

In our study, two cases with pleomorphic neoplastic cells were originally diagnosed as DLBCL but were subsequently demonstrated to be CD5- and Cyclin D1-positive in our laboratory, with a CCND1 rearrangement detected by FISH. Classical MCL is one subtype of mature B-cell lymphoma, mainly composed of small to medium lymphoid cells with irregular nuclei; however, the blastoid and pleomorphic variants of MCL can morphologically resemble LBL or DLBCL, which can cause diagnostic confusion. Therefore, it is recommended to routinely examine CD5 and Cyclin D1 for the differential diagnosis of DLBCL, blastoid/pleomorphic variant MCL, DLBCL-type Richter syndrome and primary CD5-positive DLBCL, which is more aggressive (10).

The use of insufficient immunostains can also cause the misdiagnosis of MPAL as LBL/ALL, the diagnosis of which depends heavily on the immunophenotype. There are two main approaches for MPAL typing, the European Group for the Immunological Characterization of Leukemias (EGIL) and WHO Classification criteria (1, 11). IHC can also provide helpful supplemental information for lineage specificity, especially for antibodies that located in cytoplasm and nucleus. T-cell lineage assignment is specifically relying on cytoplasmic CD3 (cCD3) expression.The confirmation of B-cell component requires multiple markers, which is based on CD19 expression (CD20 is uncommon in MPAL), corroborating with one or two other B cell makers including CD79a, PAX5 or CD22. The most specific maker of myeloid lineage assignment is MPO, together with two or more of myeloid or monocytic makers such as lysozyme, CD33, CD68, CD14. It is necessary to perform a comprehensive panel of antibodies for the diagnosis of MPAL with at least two key antibodies per lineage included.

#### 4.2.2 Poor immunostains and interpretive problems

**Figure 4:**
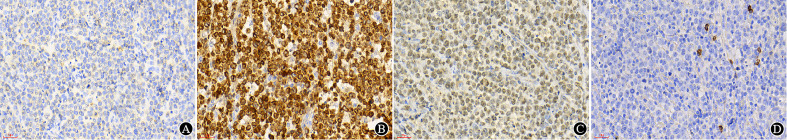
Immunostains and interpretation (×400). **(A)** In original sections made by other hospitals, CD3 staining is difficult to interpret; **(B)** After restaining in our hospital, the CD3 results were positive in the cytoplasm; **(C)** In original sections made by other hospitals, CD19 showed positive signals but was located in the nucleus; **(D)** After restaining in our hospital, CD19 was found to be negative in tumor cells, and positive signals of the internal controls were localized on the cell membrane.

An optimally immunostained slide relies on every step in tissue processing and immunohistochemical staining. It requires knowledge of the antibodies used, appropriate controls and the expected staining pattern for accurate interpretation of immunostains. Knowledge of the cellular staining location of the targeted antigen is crucial, e.g., CD20 should be located in the membrane, Cyclin D1 in the nucleus, etc., otherwise it should not be regarded as positive in any situation (**Figure 4**).

**Figure 5:**
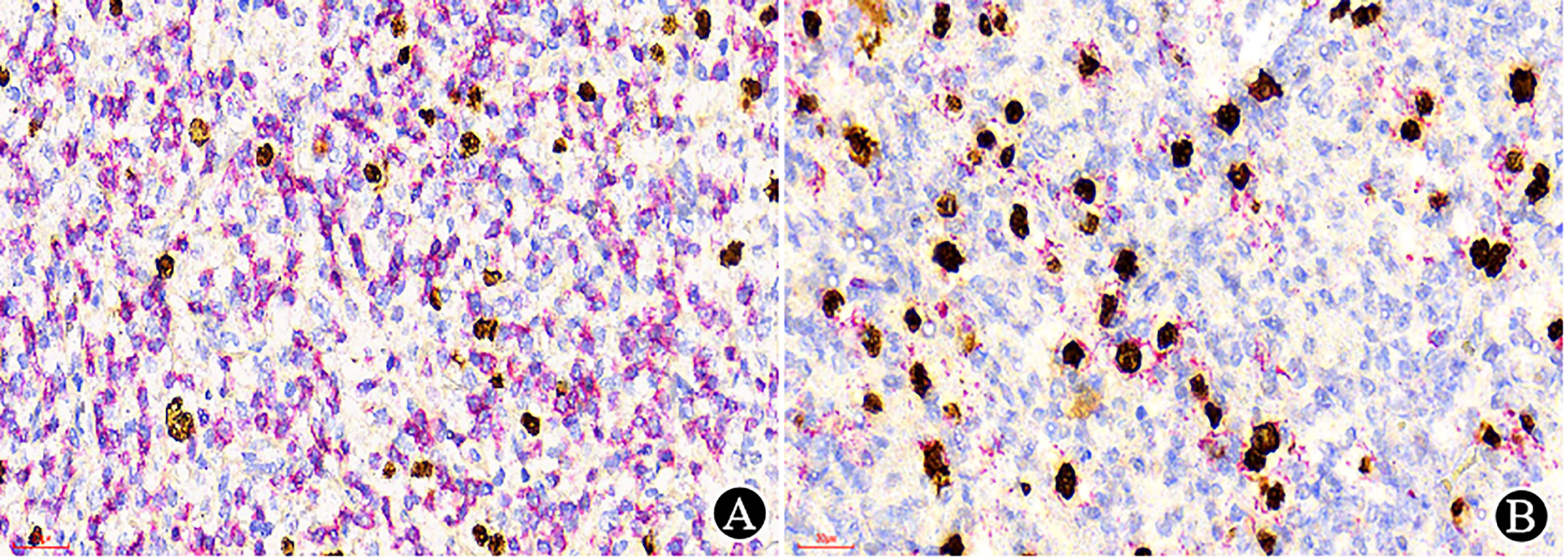
Dual-staining with EBER (nuclear stain in brown) and CD3/CD20 (membranous and cytoplasmic in red) (×400). **(A)** EBER/CD3 dual-staining showed no dual-staining positive cells; **(B)** EBER/CD20 dual-staining demonstrated dual-positive cells.

In some situations, dual-staining can be very helpful to identify the target cells (**Figure 5**) because it is useful for diagnosis or related to prognosis and treatment in some lymphomas (12).

### 4.3 Flow cytometry, molecular and cytogenetics testing in hematopathology

The importance and necessity of a comprehensive diagnosis was emphasized in the 2017 revised WHO classification. In addition to IHC, FCM and molecular and cytogenetic tests play an important role in diagnostic hematopathology.

FCM is an important tool in hematopathology due to its advantages of high sensitivity, strong specificity and short detection period. As previously mentioned, FCM has superiority in the diagnosis of MPAL, and the use of a comprehensive panel is vital to prevent misdiagnosis (13). Meanwhile, multiparameter FCM is also crucial for elucidating MPAL cases with a single population of blasts at diagnosis (biphenotypic leukaemia) or a leukaemia containing separate blast populations (bilineage leukaemia) (14, 15). In view of significant differences in clinical treatment regimens between different lineages and between immature and mature haematolymphoid malignancies, an accurate diagnosis is critical (16, 17). It is recommended that, in addition to routine histopathology and IHC, an FCM test be performed in bone marrow or extramedullary tissue as much as possible to accurately locate and analyze cell differentiation. For some special or small specimen, as for instance, FCM on brain biopsy rinse fluid is a potentially useful strategy for multidisciplinary diagnosis of central nervous system (CNS) lymphoma (18, 19).

Molecular testing is necessary for some cases. PCR-based IG and TCR gene rearrangements are important in the diagnosis of haematolymphoid tumors (20). At the same time, next-generation sequencing (NGS), which has been widely used, shows its superiority in the diagnosis, prognosis and treatment evaluation of lymphoma (21). Significant lymphoid proliferation in the lesion gastric mucosa sometimes makes it difficult to identify MALToma on the basis of morphology and immunohistochemistry only; however, IG gene rearrangement is necessary for the differential diagnosis. Based on the additional PCR-IG gene rearrangement, 11 cases modified the diagnoses from benign/reactive to MALToma, 9 cases from MALToma to benign/reactive in this study. The diagnosis of gastric MALToma should be made with caution because approximately 90% of gastric MALToma cases are related to *Helicobacter pylori (H. pylori)* infection, and approximately 50% to 90% of cases can achieve complete remission after receiving antibiotic eradication therapy (22). It should be noted that IG and TCR gene rearrangement may have lineage crossover. For example, IG rearrangement may occur in some cases of AITL, and immature haematolymphoid malignancies can often have cross-lineage rearrangements (20, 23). Nevertheless, the molecular results should be interpreted on the basis of histopathology, and attention should be given to the possibility of false negative or positive results.

FISH can provide diagnostic and prognostic prediction information by detecting specific gene sequence amplification, deletion and gene rearrangement on chromosomes in tumor tissue. The 2017 revised WHO classification proposes a new independent subtype, high-grade B-cell lymphoma (HGBL). Regardless of HGBL-NOS or HGBL with MYC, BCL2 and/or BCL6 rearrangements (double/triple-hit lymphoma), the detection of MYC, BCL2 and BCL6 by FISH is necessary for a clear diagnosis (1).

For newly diagnosed patients, it is recommended that an MICM (Morphology, Immunology, Cytogenetics and Molecular) examination be as thorough as possible to provide comprehensive data for monitoring and follow-up.

### 4.4 Attention to clinical information

Accurate clinical information (including gender, age, symptoms, and auxiliary examinations) is indispensable for pathological diagnosis. Some studies suggest that clinical information accounts for approximately 10% of the information required for the diagnosis of lymphomas (24). The diagnosis of DLBCL in children should exclude BL and B-LBL/ALL first. It is necessary to rule out skeletal lesions in the diagnosis of extraosseous (extramedullary) plasmacytomas, and a clear mass is a prerequisite for the diagnosis of primary DLBCL of the uterine cervix. The 2017 revised WHO lists pediatric-type follicular lymphoma (PTFL) as an independent subtype of B-NHL. PTFL primarily involves the lymph nodes of the head and neck with localized lesions, and its pathological grade is usually FL-3A in morphology. Its immunophenotype and genetic characteristics overlap with those of common FL, but its clinical manifestations are different from those of common FL (1, 25). Whether a localized lesion is found by clinical evaluation is crucial for the diagnosis of PTFL. This is a rare type of lymphoma with a better prognosis, and the clinical treatments are significantly different from those for common FL (25, 26). Therefore, attention should be given to clinical information to improve the accuracy of diagnosis.

### 4.5 Pathologists’ subjectivity and diagnostic challenges in hematopathology

Haematolymphoid neoplasms have a wide variety and complex classification. With the rapid development of new technologies such as FCM, cytogenetics, and molecular pathology, the understanding of haematolymphoid neoplasms has been further deepened. Despite the application of innovative technologies, diagnosis in hematopathology is sometimes difficult. Meanwhile, due to variations in knowledge, training background and practical experience, pathologists’ subjective judgments can lead to different conclusions from the same specimen.

#### 4.5.1 HGBL vs. DLBCL/BL

HGBL was proposed as an independent subtype in the 2017 revised WHO classification, including 1. HGBL with MYC, BCL2 and/or BCL6 rearrangements, i.e., double/triple-hit lymphoma; 2. HGBL-NOS. In this study, 54 cases were corrected to HGBL, and most of the original diagnoses were DLBCL or BL. One more thing is that some pathologists can confuse the definition of HGBL with just simply highly aggressive lymphoma. This suggests that pathologists should strictly grasp the diagnostic criteria of HGBL, including the morphology and immunophenotype; furthermore, FISH is necessary to detect MYC, BCL2 and BCL6 rearrangements. For patients who were diagnosed with DLBCL or BL, MYC, BCL2, and BCL6 rearrangements should be detected by FISH to exclude double/triple-hit lymphoma. What is noteworthy is that MYC rearrangement is found not only in BL and HGBL with MYC and BCL2 and/or BCL6 rearrangements but also in DLBCL, FL, PBL, B-LBL/ALL, and even in myeloid tumors (27, 28).

#### 4.5.2 Low-grade FL vs. high-grade FL vs. DLBCL

FL is a common subtype of B-cell lymphoma, which can be divided into low-grade FL (grades 1 and 2) and high-grade FL (grades 3A and 3B) according to the WHO classification. The pathological grading of FL should be accurate, since low-grade and some grade 3A FL patients have indolent clinical features and can be followed up or treated locally, while grade 3B FL patients should be treated according to the treatment strategy of DLBCL (29). Pathologists should be strict and proficient in the diagnosis and grading criteria of FL and accurately identify central cells (small, with little cytoplasm and irregularly angulated or cleaved nuclei) and centroblasts (large in size, with round or oval nuclei and 1 to 3 peripheral nucleoli), especially for cases of morphological variation. Misdiagnosis between low-grade and high-grade tumors may result in excessive or delayed treatment. Moreover, Ki-67 cannot be used as a grading factor for FL, but low-grade FL with high proliferative activity (Ki-67 ≥ 30%) is often considered to be clinically aggressive, with a risk of transformation to high-grade FL (30).

#### 4.5.3 CHL vs. NHL

In most cases, distinguishing CHL from NHL seems to be relatively easy, but the diagnostic boundary can sometimes be difficult to discern. In this study, the misdiagnoses of CHL included benign granulomatous hyperplasia, thymoma, DLBCL, and T-NHL. Some subtypes of PTCLs, especially those with a follicular helper T-cell (TFH) phenotype, can share similar morphology and immunophenotype, including a complex inflammatory background of cell proliferation and Hodgkin/Reed-Sternberg (HRS) cells that present features of CHL phenotypes such as CD30 positivity, PAX5 weak positivity, CD45 negativity, and EBER positivity, leading to a misdiagnosis between them (31–34). However, the properties of proliferated T cells are completely different: T cells in AITL or PTCL with the TFH phenotype are malignant, while T cells in CHL are reactive. TCR rearrangement is useful for the differential diagnosis. In view of completely different treatments and prognoses, the diagnosis should be made with as much caution as possible. It should be noted that HRS cells are also observed in small B-cell lymphomas such as SLL/CLL, FL, and MCL (35). In the differential diagnosis of CHL and DLBCL, in addition to morphological differences, IHC of PAX5, CD20, CD45 (LCA), BOB1, and OCT2 can be helpful.

#### 4.5.4 Castleman disease vs. other reactive lymphadenopathies

**Figure 6:**
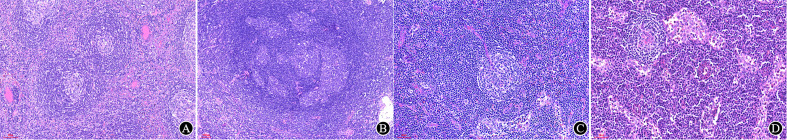
Typical Castleman disease. **(A)** Follicles with atrophic germinal centers and expanded mantle zones, forming a concentric annular structure, with an “onion-skin” look (×150); **(B)** Multiple germinal centers within a single mantle area (×65); **(C)** A remarkable hyalinized vessel grows into the germinal centers, forming a “lollipop” appearance (×200); **(D)** Sheets of plasma cells are seen in the interfollicular region in PC-type CD (×200).

Castleman disease (CD) (**Figure 6**) should be distinguished from other reactive lymphadenectasis since a few could develop into tumors (36, 37). The clinical and pathological manifestations of CD are so heterogeneous that they are easily confused with other diseases that can cause lymphadenopathy, such as infectious diseases, autoimmune diseases, and tumors. In some difficult cases, the pathologist can communicate with clinicians and give a descriptive diagnosis that is acceptable to both parties and does not mislead clinical treatment.

### 4.5.5 Haematolymphoid neoplasms vs. non-haematolymphoid neoplasms

**Figure 7:**
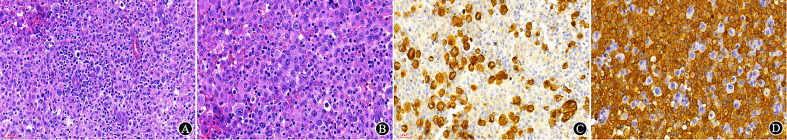
Misdiagnosed metastatic carcinoma. **(A, B)** In HE sections, there was significant histiocytic proliferation, atypical cells with large nuclei, and unremarkable epithelioid structure (A ×200, B ×300); **(C)** CKpan staining was positive in tumor cells (×300); **(D)** CD163 staining was positive in histiocytes (×300).

Non-haematolymphoid neoplasms are also one of the common differential diagnoses for haematolymphoid neoplasms (30 cases in this group, accounting for 3.3% of misdiagnosed cases, 1.31% of all cases). Some poorly differentiated carcinomas, sarcoma and neuroendocrine tumors can manifest as small round cells morphologically resembling lymphoma. In this study, one lymph node biopsy demonstrated only PAX5, which was weakly positive in the T/B lineage markers, but showed CKpan, Syn and CD56 positivity in subsequent immunostains; a neuroendocrine carcinoma was subsequently diagnosed. What should be considered is that PAX5 can be expressed not only in B lymphocytes but also in some neuroendocrine carcinomas and rhabdomyosarcomas (38). It was reported that CD138, MUM-1 and CD56 can also be expressed in malignant melanoma (8), which can lead to a misdiagnosis of plasmacytoma; moreover, CD99 is expressed not only in Ewing’s sarcoma but also in precursor lymphocytes (39). There was another case of such misdiagnosis in our study. The patient attended a clinical consultation because of a mass on the upper arm, and tissue biopsy showed significant histiocytic proliferation. A histiocytic sarcoma or epithelioid sarcoma was suspected in several Class A tertiary hospitals. However, there were scattered atypical cells with CD163 and CD68 negativity against the background of a large number of proliferated histiocytes. Then, the markers CKpan, CK7 and TTF1 were stained, and a metastatic carcinoma was confirmed (**Figure 7**). The patient subsequently underwent a pulmonary lobectomy, and a poorly differentiated adenocarcinoma of the lung with partial large cells was indicated in the postoperative pathology. This patient was first admitted for metastatic carcinoma of the upper arm, and the biopsy showed a large number of proliferated histiocytes, which made it difficult to distinguish the mass from histiocytic sarcoma or other epithelioid sarcomas. Histiocytic sarcoma is rare. Pathologists should be cautious, should exclude the possibility of common tumors first and then should consider the rare types.

Finally, pathologists should try their best to give a clear diagnosis and avoid too many tendentious diagnoses.

## 5 Conclusion

This study showed that the misdiagnosis rate of haematolymphoid neoplasms is still high in China and involves various types of misdiagnosis and complicated causes. The hematopathology is more challenging than that in other subspecialties of pathology. Therefore, pathologists should not only be proficient in morphology and immunohistochemistry but must also remain updated regarding advancements in technology; this, integrated the clinical information and ancillary data formulating a diagnosis as accurate as possible based on the latest WHO classification to ensure timely, standardized, precise and individualized treatment for patients. Meanwhile, the systematic specialized training of pathologists in hematology is recommended to enhance their cognition in this specialty and improve the diagnosis level of hematopathology in China.

## Data availability statement

The original contributions presented in the study are included in the article/supplementary material. Further inquiries can be directed to the corresponding author.

## Ethics statement

The studies involving human participants were reviewed and approved by ethical committee of Beijing GoBroad Boren Hospital. The patients/participants provided their written informed consent to participate in this study.

## Author contributions

JD wrote the article, collected the materials. XZ performed the statistical analysis. LY performed the experiments. ZG and CZ reviewed the cases. LG designed the paper and revised the paper. All authors contributed to the article and approved the submitted version.
